# From monkey single-cell atlases into a broader biomedical perspective

**DOI:** 10.1093/lifemedi/lnac028

**Published:** 2022-08-11

**Authors:** Xuanxuan Zou, Xi Dai, Alexios-Fotios A Mentis, Miguel A Esteban, Longqi Liu, Lei Han

**Affiliations:** BGI-Shenzhen, Shenzhen 518103, China; College of Life Sciences, University of Chinese Academy of Sciences, Beijing 100049, China; BGI-Shenzhen, Shenzhen 518103, China; College of Life Sciences, University of Chinese Academy of Sciences, Beijing 100049, China; BGI-Shenzhen, Shenzhen 518103, China; Laboratory of Integrative Biology, Guangzhou Institutes of Biomedicine and Health, Chinese Academy of Sciences, Guangzhou 510530, China; BGI-Shenzhen, Shenzhen 518103, China; College of Life Sciences, University of Chinese Academy of Sciences, Beijing 100049, China; BGI-Shenzhen, Shenzhen 518103, China

The cell is the fundamental constituent unit of a mammal which, in turn, depends on the cooperative work of various cells to compose intricate life activities. Cells harbor diverse features, such as genome sequence (including genetic mutations that may be present only in cellular subpopulations), epigenetic changes (including chromatin status and histone or epitranscriptomic modifications) as well as protein abundance, and localization, while they are also guided by cellular-programming processes. In doing so, cells exhibit distinct phenotypes and form a variety of functional tissues and organs, building up every part of life; of note, all mammals go through this process from birth to death. To better understand both biological and pathological mechanisms of a variety of tissues, it is crucial to construct comprehensive cell atlases of various species by harnessing techniques which can profile—at the quantitative level, from a wide span of organ systems, and at a previously unprecedented detail—gene expression and chromatin state; such techniques are currently described as ultrahigh-throughput single-cell sequencing technology (Fig. 1A). Doing so is of paramount importance toward advancing our knowledge about complex but not yet fully deciphered biological problems concerning mammals but also toward developing advanced diagnostic and targeted therapeutic tools against several pathologies.

**Figure 1.**
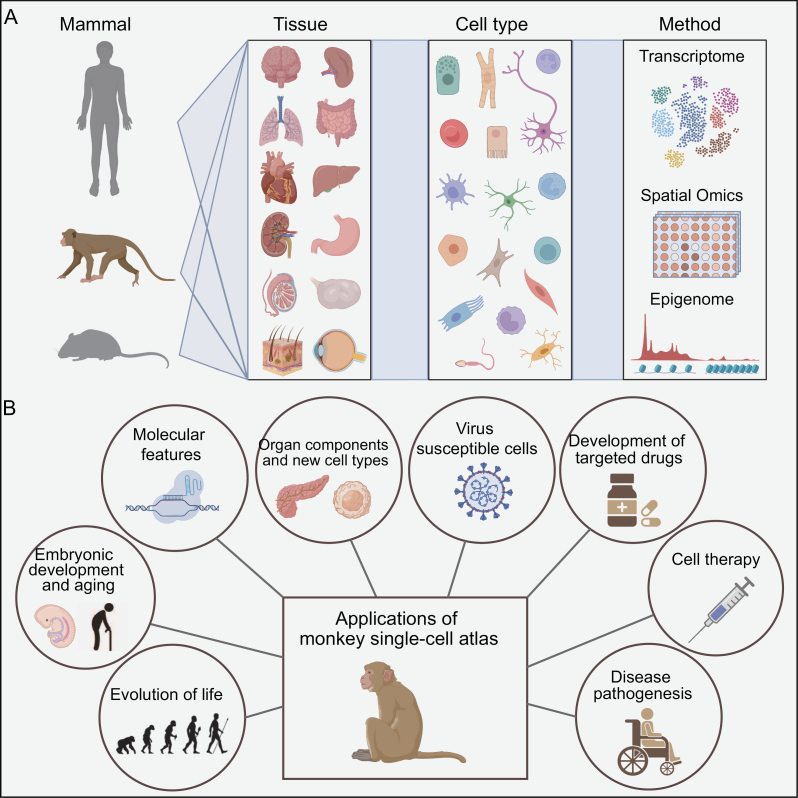
(A) A variety of single-cell methods have been applied to construct single-cell atlases of mammals to reveal the cellular heterogeneity of corresponding cell types in different tissues. (B) Potential future applications of monkey single-cell transcriptomic atlas.

Recent years, a burst of remarkable advances has been noted in the construction of cell atlas using single-cell sequencing technology. To begin with, the Human Cell Atlas Consortium accumulated a voluminous amount of single-cell transcriptomic data based on an analysis of major human tissues and organs [1, 2]. In addition, large-scale transcriptome and chromatin accessibility atlases of mice and humans at all body tissues’ level have also been delineated, respectively. These studies provide us with further insights into mammalian cellular heterogeneity, as well as the composition and function of cell types in corresponding tissues. However, there are considerable settings (notably, human diseases) where it is rather difficult to directly conduct experimentation by obtaining human tissue samples for several reasons; the latter range from bioethical dilemmas and difficulties accessing these issues to scarcity of tissue availability. In parallel, traditional experimental animal models, such as mice, do not always constitute the ideal approach to study human development and diseases, due to their considerable genetic differences with humans, especially when regarding neurological and mental health disorders. Recently, a large-scale single-cell transcriptome atlas of cynomolgus macaque covering 45 organs and characterizing 113 major cell types has been the basis to create an ultrahigh-resolution cell map, which supplies broad and crucial reference data for both human and nonhuman primate research [3]. Meanwhile, a single-cell atlas depicting 27 major tissues/organs of mouse lemur (*Microcebus murinus*), a model species that is equally widely used, has also been recently reported [4]. Collectively, these single-cell data can provide vital data resources for researchers working on primates, whose findings can then be extrapolated to human pathophysiology.

Single-cell transcriptomic atlases have been shown to facilitate the discovery of new cell types, especially the rare ones. Besides, this approach reveals species differences in cell features, and it enables the inference of cell types that are linked to several disease statuses, such as in genetic, infectious, or cardiovascular disorders. For instance, Ezran et al. found 10 previously unrecognized or sparsely characterized cell types and subtypes in *Tabula Microcebus* [4], whereas Han et al. found some precursor cells expressing stem cell markers in adult tissues, which may provide a cell source for cell therapy within subsequent repair of organ and/or cell damages [3]. To illuminate species differences at cell resolution, Quake et al. explored the phylogenetic evolution of primate cell types by comparing mouse lemur cell repertoires with their homologous cells in humans, revealing cell type-specific patterns of primate cell specialization [4]. Meanwhile, Han et al. performed a series of comparisons in homologous cell features, cell–cell interactions, and genetic diseases across mice, monkeys and humans. As expected, some human neurological genetic traits or diseases are highly linked to the same cell type of human and monkey species but rarely linked to the corresponding cell type of mice. For example, schizophrenic traits were strongly associated with cortical excitatory neurons in primates but not in mice [3]. These findings indicate that monkey—such as cynomolgus macaque, which is one of the most popular nonhuman primates—are a more suitable experimental proxy to study human disease studies than mice. Moreover, we can infer possible cellular mechanisms of pathogenesis by applying the macaque cell atlas data set. For example, regarding infectious diseases, Han et al. applied the cell atlas to construct a virus database containing 126 virus-susceptible cell types, by which the kind of cells that are most likely to be infected by a specific virus can be easily queried [3]. In addition, by constructing cellular atlases of the lung and cardiovascular system of young and old primates, Ma et al. revealed molecular pathways associated with either impaired primary cellular functions during aging or compromised cellular intrinsic host defense while, in parallel, they dissected the cellular and molecular basis of vulnerability to age-related diseases and COVID-19 [5]. Besides connecting human genetic diseases with their associated genes, single-cell transcriptomic atlases provide links between genetic risk variants and cells possibly implicated in the expression of these variants at the RNA level. Human phenotypic characteristics can also be mapped to the cell types they may refer to [6]. Therefore, cell maps could help promoting precision medicine (at either the prevention and/or treatment level) in the future by linking human genetic traits and indexing disease-associated cells. More specifically, cell maps could also provide data-driven support to elucidate the pathogenesis of monogenic and, moving forward, even complex/polygenic genetic diseases, so as to (i) conduct studies related to drug evaluation and screening, (ii) assist to the development of targeted drugs, and (iii) give basic resources and tools for the production of novel biomedicines [1]. As for aging-related phenotypes, cynomolgus macaque is also a preferred animal model for reproductive toxicity studies given its menstrual and ovarian cycle’s similarity to that of human females. In that context, Wang et al. generated a single-cell transcriptomic atlas of ovarian aging in cynomolgus macaque, and they demonstrated that oxidative damage is a key factor causing the decline in ovarian function with age [7]. Subsequently, single-cell transcriptome atlases of the aging process of arteries and hippocampus were also constructed [8]. These atlases have provided much valuable insights for corresponding developmental processes of crucial human organs and tissues that are linked to major human diseases, such as atherosclerosis and Alzheimer’s disease, respectively. Therefore, on the basis of single-cell transcriptomic atlases, novel avenues for new disease and treatment-testing experimental models are expected to be further opened. On the whole, these large-scale monkey cell atlases are essential for the scientific understanding of species evolution, embryonic development, organ structure composition, as well as aging, and human diseases (Fig. 1B) [1, 3, 5, 6].

Invigoratingly, the continuous development of single-cell sequencing technology pertains to all core yet exponentially increasing aspects of this technology, i.e. ranging from the throughput and the sequencing depth to the capture efficiency of single-cell sequencing, as well as the technological and bioinformatics capacity for analyzing sequencing data from distinct kinds of species. In addition, the quantity of cells and the range of tissues and organs covered, the identifiable types of cells, the number of genes detected, and the sequencing depth of single-cell RNA and ATAC atlases have also undergone dramatic improvements. Therefore, it comes as a logical expectation that future single-cell atlas projects will identify more sophisticated cell types and states, incorporating all tissues and organs, compatible to more species. Doing so can enable us to (i) discover and characterize previously unknown cell types but also ontogenetically intermediate cellular states, (ii) enable researchers to obtain a profound understanding of cellular growth, migration, and interactions, and (iii) assist to clarify how different cell types function and respond to diseases-induced disrupted homeostasis.

As known, manifold cell types are distributed in different spatial locations. Conversely, when isolated from specific spatial locations and micro-environments, they lose interaction with neighbor cells, making it difficult for them to serve intercellular functions. In recent years, several tangible experimental results have been produced on spatial transcriptomics, and multiple corresponding spatiotemporal atlases of many species have been established [9]. Such approaches empower us to obtain transcriptional information at different spatial locations and to track the spatial distribution of different cell types.

In fact, a key area of immediate interest is oncology in which the high heterogeneity among tumor cells and the characteristics of clonal evolution in the process of tumor occurrence, development, and treatment can be studied. By studying the transcriptomics and epigenetics features of the cell atlas, single-cell approaches can be used to monitor the progress, curative effect, and prognosis of malignancies, and discover therapeutic targets to guide the clinical medication. Another area of potential interest is molecular syndromology which aims to offer bridges between clinical genetics and developmental biology through functional genomics (e.g. by studying variant function in zebrafish, mice, and so on). Besides studying the development trajectory of ontogeny-implicated genes, exploring through single-cell omics approaches how clinically derived variants from the above genes lead to congenital malformations and syndromes can offer personalized molecular modeling from patients. Another potential future focus is neurological and neuropsychiatric disorders. Given the multitude of genes implicated in brain development and, in turn, the myriads of variants linked to the psychosis spectrum, applying single-cell omics approaches to studying induced pluripotent stem cells derived from patients in question could provide molecular clues that could affect treatment options, in a way more sophisticated than traditional next-generation sequencing.

All things considered, cell atlases are expected to be the fine-tuned *radar* for assessing any types of cells in the long term. They aid us to gain a better grasp of cellular migration, cellular differentiation, and cell-to-cell interaction, and they explain the spatial heterogeneity that may be unique to some cell types (Fig. 1A). Moving forward, through the rapid technological progressions, the combination of using more samples from different developmental states will also enable us to get a comprehensive spatiotemporal multidimensional cell map of mammals. In this view, single-cell sequencing technology appears as a core technological solution to advance precision medicine. Collectively, such approaches can optimize precision diagnostics and treatment of diseases.

## References

[1] Liu Z, Zhang Z. Mapping cell types across human tissues. Science 2022;376:695–6. 35549410 10.1126/science.abq2116

[2] Zhong S, Ding W, Sun L, et al. Decoding the development of the human hippocampus. Nature 2020;577:531–6. 31942070 10.1038/s41586-019-1917-5

[3] Han L, Wei X, Liu C, et al. Cell transcriptomic atlas of the non-human primate *Macaca fascicularis*. Nature 2022;604:723–31. 35418686 10.1038/s41586-022-04587-3

[4] Consortium TTM. Tabula Microcebus: a transcriptomic cell atlas of mouse lemur, an emerging primate model organism. bioRxiv 2021, preprint: not peer reviewed. 10.1101/2021.12.12.469460.

[5] Ma S, Sun S, Li J, et al. Single-cell transcriptomic atlas of primate cardiopulmonary aging. Cell Res 2021;31:415–32. 32913304 10.1038/s41422-020-00412-6 PMC7483052

[6] Eraslan G, Drokhlyansky E, Anand S, et al. Single-nucleus cross-tissue molecular reference maps toward understanding disease gene function. Science 2022;376:eabl4290. 35549429 10.1126/science.abl4290 PMC9383269

[7] Wang S, Zheng Y, Li J, et al. Single-cell transcriptomic atlas of primate ovarian aging. Cell 2020;180:585–600. 32004457 10.1016/j.cell.2020.01.009

[8] Zhang H, Li J, Ren J, et al. Single-nucleus transcriptomic landscape of primate hippocampal aging. Protein Cell 2021;12:695–716. 34052996 10.1007/s13238-021-00852-9 PMC8403220

[9] Chen A, Liao S, Cheng M, et al. Spatiotemporal transcriptomic atlas of mouse organogenesis using DNA nanoball-patterned arrays. Cell 2022;185:1777–92. 35512705 10.1016/j.cell.2022.04.003

